# Binocular Summation and Suppression of Contrast Sensitivity in Strabismus, Fusion and Amblyopia

**DOI:** 10.3389/fnhum.2019.00234

**Published:** 2019-07-12

**Authors:** Michael Dorr, MiYoung Kwon, Luis Andres Lesmes, Alexandra Miller, Melanie Kazlas, Kimberley Chan, David G. Hunter, Zhong-Lin Lu, Peter J. Bex

**Affiliations:** ^1^Department of Electrical and Computer Engineering, Technical University Munich, Munich, Germany; ^2^Department of Ophthalmology, School of Medicine, University of Alabama at Birmingham, Birmingham, AL, United States; ^3^Adaptive Sensory Technology, San Diego, CA, United States; ^4^College of Medicine and Health, University of Exeter, Exeter, United Kingdom; ^5^Department of Ophthalmology, Boston Children’s Hospital, Harvard Medical School, Boston, MA, United States; ^6^Department of Ophthalmology, Harvard Medical School, Boston, MA, United States; ^7^Department of Psychology, The Ohio State University, Columbus, OH, United States; ^8^Department of Psychology, Northeastern University, Boston, MA, United States

**Keywords:** amblyopia and strabismus, contrast sensitivity function (CSF), quick CSF, visual acuity, binocular summation

## Abstract

**Purpose**: Amblyopia and strabismus affect 2%–5% of the population and cause a broad range of visual deficits. The response to treatment is generally assessed using visual acuity, which is an insensitive measure of visual function and may, therefore, underestimate binocular vision gains in these patients. On the other hand, the contrast sensitivity function (CSF) generally takes longer to assess than visual acuity, but it is better correlated with improvement in a range of visual tasks and, notably, with improvements in binocular vision. The present study aims to assess monocular and binocular CSFs in amblyopia and strabismus patients.

**Methods**: Both monocular CSFs and the binocular CSF were assessed for subjects with amblyopia (*n* = 11), strabismus without amblyopia (*n* = 20), and normally sighted controls (*n* = 24) using a tablet-based implementation of the quick CSF, which can assess a full CSF in <3 min. Binocular summation was evaluated against a baseline model of simple probability summation.

**Results**: The CSF of amblyopic eyes was impaired at mid-to-high spatial frequencies compared to fellow eyes, strabismic eyes without amblyopia, and control eyes. Binocular contrast summation exceeded probability summation in controls, but not in subjects with amblyopia (with or without strabismus) or strabismus without amblyopia who were able to fuse at the test distance. Binocular summation was less than probability summation in strabismic subjects who were unable to fuse.

**Conclusions**: We conclude that monocular and binocular contrast sensitivity deficits define important characteristics of amblyopia and strabismus that are not captured by visual acuity alone and can be measured efficiently using the quick CSF.

## Introduction

Amblyopia and strabismus are the most common developmental disorders of binocular vision, with an estimated prevalence of around 2%–5% (Graham, 1974; Ross et al., 1977; Friedmann et al., 1980; Cohen, 1981; Simpson et al., 1984; Stayte et al., 1990; Thompson et al., 1991; Satterfield et al., 1993; Kvarnström et al., 2001; Jakobsson et al., 2002; Barry and König, 2003; Robaei et al., 2005). Amblyopia affects the spatial vision of one or both eyes in the absence of an obvious organic cause and is associated with a history of abnormal visual experience during development (The Lasker/IRRF Initiative for Innovation in Vision Science, 2018). Strabismus impairs the ability to align the eyes so that targets are imaged on the fovea of the *fixing* eye and on parafoveal retina in the strabismic eye. The magnitude of strabismus may vary with viewing distance, such that some people with strabismus are able to fuse at some viewing distances (Hatt et al., 2008), and the strabismic deviation may be constant or intermittent (for review, see Helveston, 2010). Strabismus is associated with social difficulties (Satterfield et al., 1993; Olitsky et al., 1999; Jackson et al., 2006), a reduced quality of life (Tandon et al., 2014), elevated risk of sustaining musculoskeletal injury, fracture, or fall (Pineles et al., 2015b), and a negative impact on employment opportunities (Goff et al., 2006; Mojon-Azzi and Mojon, 2007).

Strabismus is a common cause of amblyopia (Woodruff et al., 1994b; Simons, 2005), although amblyopia can also be caused by other developmental disorders such as anisometropia or visual deprivation (Helveston, 2010). Amblyopia is associated with deficits in spatial vision (Robaei et al., 2005; Zhao et al., 2017) including reduced visual acuity (Kirschen and Flom, 1978; Levi and Klein, 1982, 1985; Kelly and Buckingham, 1998), contrast sensitivity loss (Hess and Howell, 1977; Levi and Harwerth, 1978; Bradley and Freeman, 1981; Kiorpes et al., 1999; McKee et al., 2003), spatial distortion (Pugh, 1958; Hess et al., 1978; Fronius and Sireteanu, 1989; Hess, 2001), abnormal contour integration (Hess et al., 1997; Hess and Demanins, 1998), and binocular acuity summation (Sireteanu, 1982; Chang et al., 2017) deficits.

The current standard treatment for amblyopia is to provide a period of refractive correction (Cotter et al., 2012), then to use occlusion (eye patching) or penalization (blurring eye drops) therapies that temporarily impair vision in the fellow eye and force the use of the amblyopic eye (for review, see Clarke, 2010). Strabismus may be treated surgically (Mets et al., 2004), with prism correction (Gunton and Brown, 2012), or with vision therapy (Scheiman et al., 2011; for review, see Rutstein et al., 2012). However, these treatments for amblyopia (Woodruff et al., 1994a; Pediatric Eye Disease Investigator Group, 2003; Fresina and Campos, 2014) and strabismus (Fresina and Campos, 2014) rarely restore normal binocular vision. Consequently, after treatment, many patients experience persistent interocular suppression (Holopigian et al., 1988; Hess, 1991; Harrad, 1996; Kwon et al., 2015) or deficits in stereoacuity (Stewart et al., 2013; Levi et al., 2015) and binocular acuity summation (Blake and Fox, 1973; Chang et al., 2017).

Visual acuity is the main clinical measure for functional outcomes. However, many studies have shown that contrast sensitivity remains impaired in the affected eye even after normal acuity has been achieved by amblyopia treatment (Regan et al., 1977; Sjöstrand, 1981; Rogers et al., 1987; Cascairo et al., 1997; Huang et al., 2007). In many cases, visual acuity deficits may be more evident with low- than high-contrast visual acuity tests (Pineles et al., 2013, 2014b, 2015a). Furthermore, the contrast sensitivity loss in amblyopia is spatial-frequency dependent, a property that cannot be assessed by high- or low-contrast visual acuity alone. In several studies the amblyopic eye showed reduced contrast sensitivity that mostly occurred at mid-high spatial frequencies, while deficits at low spatial frequencies were less common (Hess and Howell, 1977; Levi and Harwerth, 1977; Rentschler et al., 1980; Sjöstrand, 1981; Howell et al., 1983), suggesting a significant need for assessing spatial-frequency dependent deficits in characterizing amblyopic vision.

Although the contrast sensitivity function (CSF) of the fellow fixing eye remains normal or near normal in amblyopia (Cascairo et al., 1997), binocular summation is greatly compromised or absent in amblyopia (Levi et al., 1980; Sireteanu et al., 1981; Pardhan and Whitaker, 2000; Hess et al., 2014) unless the sensitivity deficit of the amblyopic eye is compensated (Pardhan and Gilchrist, 1992; Baker and Meese, 2007; Baker et al., 2007a). In principle, the binocular summation deficit could arise from impaired mechanisms of binocular interaction that are not spatially-selective (Huang et al., 2011). Alternatively, it may depend on structural correlations, which may show spatial frequency selective effects of inter-ocular refractive differences, as in anisometropia, Holopigian et al. (1986) or misaligned binocular receptive fields, as in strabismus (Thorn and Boynton, 1974). In subjects with intermittent strabismus, binocular summation may, therefore, depend on whether fusion is possible at the testing distance. These spatial-frequency dependent features of contrast sensitivity deficits make the CSF a good candidate for evaluating monocular and binocular vision in strabismus and amblyopia.

While the need for effective assessment of contrast deficits in the patient population has been recognized (Owsley and Sloane, 1987; Sebag et al., 2016) its clinical assessment has been frustrated due to the long testing times of psychophysical assessments (Mansouri et al., 2008). To examine the role of amblyopia and strabismus in binocular contrast summation, we therefore measured the full monocular and binocular CSFs with the quick CSF method (Lesmes et al., 2010; Dorr et al., 2013) in amblyopes, subjects with strabismus but not amblyopia (who were either able or not to fuse at near test distances), and normally-sighted controls.

## Methods

### Participants

The study design included three groups: patients with: (1) strabismic, anisometropic, or mixed amblyopia (AMB); (2) strabismus without amblyopia (SWA); and (3) normally sighted individuals (NSC). Inclusion and exclusion criteria were:

1. Clinical amblyopia is often defined as at least 0.2 logMAR interocular difference in acuity with best correction. Here, we adopted the clinical definition of amblyopia for our inclusion and exclusion criteria. Strabismic amblyopia was defined as a ≥0.2 logMAR interocular difference in best-corrected visual acuity (BCVA). Strabismus was defined as angular deviation between eyes of 5–50 prism diopters at either near or far viewing distances. Anisometropic amblyopia was defined as a 2-line or greater interocular difference (≥0.2 logMAR) in BCVA with tropia ≤4 prism diopters.
2. Strabismus without amblyopia was defined as ≤0.2 logMAR difference between the monocular BCVAs. Strabismus was defined as above. Intermittent strabismus with near fusion was defined as ≤4 prism diopters at 40 cm test distance.
3. Normal vision was defined as BCVA ≤0.0 logMAR or uncorrected VA ≤0.2 logMAR for both eyes without any latent or manifest ocular deviation other than phorias within normal limits.
4. Subjects with any known cognitive or neurological impairments were excluded.

Informed consent was obtained from subjects or (in addition to subjects’ assent) from the parents or legal guardian of subjects aged <18 years, in accordance with procedures approved by the IRB of Boston Children’s Hospital and complying with the Declaration of Helsinki. Enrolled patients underwent complete clinical examination, including best corrected visual acuity (ETDRS charts, letter-by-letter scoring was used), cycloplegic refractive error, stereopsis (Titmus Fly SO-001), ocular motility, binocular fusion (a Worth 4 dot test) and cover test at near and distance fixation. The angle of any heterotropia or heterophoria was measured by prism-and-cover test at near and distance fixation. We only report the measurements made at near fixation, which is relevant to the 60 cm viewing distance of CSF assessment. Minimum participant age was 5 years and all participants were able to perform letter acuity assessment. Participant characteristics are provided in Table 1. All subjects were tested with best-corrected vision in the CSF test; as can be seen in Table 1, there was some overlap of visual acuities between the groups (i.e., one out of 11 amblyopic eyes had better acuity than three normal eyes but with interocular difference ≥0.2 logMAR). For consistency across groups, hereafter we term the amblyopic eye and fellow eye as *the non-dominant eye (NDE)* and *dominant eye (DE)*, respectively. The DE was determined by the results from clinical binocular function or acuity test (for AMB and SWA subjects) or finger pointing task (for NSC).

**Table 1:** Participant characteristics.

			Amblyopia	Strabismus	Normal
			(*N* = 11)	(*N* = 20)	(*N* = 24)
Age (years)	mean (±SD)		15.9 (±16.2)	19.9 (±20.4)	18.7 (±10.8)
	min:median:max		6:9:50	5:9.5:68	5:17:43
Gender	ratio (female:male)		5:6	13:7	11:13
Visual Acuity (logMAR)	mean (±SD)	non-dominant eye	0.55 (±0.35)	0.09 (±0.11)	0.01 (±0.08)
		dominant eye	0.02 (±0.09)	0.05 (±0.09)	−0.04 (±0.08)
	min:median: max	non-dominant eye	0.14:0.48:1.30	−0.08:0.12:0.30	−0.12:0:0.18
		dominant eye	−0.12:0:0.18	−0.10:0.02:0.20	−0.22/−0.02/0.10
Angular Deviation (prism diopter)	mean (±SD)		10.2 (±14.8)	17.3 (±14.0)	Neither manifest
					nor latent
					deviation
	min:median:max		0:4:45	4:10:50	
Ability to fuse			*N* = 5	*N* = 7	*N* = 24
Strabismus type (intermittent)			NA	esotropia 7 (0)	NA
				exotropia 6 (4)
				esophoria 4 (2)
				exophoria 3 (0)

### Procedure

Spatial CSFs were assessed with the quick CSF method (Lesmes et al., 2010) and a 10-AFC letter recognition task (Hou et al., 2015) implemented on a tablet computer (Dorr et al., 2013). The quick CSF method is a Bayesian adaptive procedure that takes advantage of prior knowledge about the general shape of the CSF and searches the 2D stimulus space (contrast and spatial frequency) to find stimuli for future trials that maximize information about the subject’s individual CSF.

The CSF maps a spatial frequency *f* to a sensitivity *S* by a truncated log-parabola *S(f, θ)* that is based on a log-parabola *S*_0_*(f,θ)*. The parameter vector Θ has four dimensions: (i) peak gain, *γ*_max_; (ii) peak spatial frequency, *f*_max_; (iii) bandwidth, *β*; and (iv) low-frequency truncation level, *δ*.

$$\log_{10}\left[S_{0}(f,\theta )\right]=\log_{10}(\gamma_{\max})-\frac{4}{\log_{12}2}{\left(\frac{\log_{10}(f)-\log_{10}(f_{\max})}{\beta }\right)}^{2}$$

$$\log_{10}\left[S(f,\theta )\right]=\{\begin{array}{c} \log_{10}(\gamma_{\max})-\delta \text{ if }f<f_{\max}\wedge \log_{10}S_{0}<\log_{10}(\gamma_{\max})-\delta \\ \log_{10}S_{0}(f,\theta )\text{ otherwise} \end{array}$$

The quick CSF also provides two important scalar features: (i) a summary statistic, the area under the log CSF (AULCSF; Applegate et al., 1998) (ii) and the high spatial-frequency cut-off (CSF Acuity). Test letters were band-pass filtered Sloan letters with peak frequency of 0.64–41 cycles per degree (cpd) at the viewing distance of 60 cm. Each of 25 trials began with a 500 ms white bounding box cueing the size and location of the upcoming stimulus. Then, the target letter was presented for 2 s followed by a response interval. The experimenter entered the subject’s response using a keyboard, which initiated a subsequent trial. No feedback was provided.

In random order, the CSF was measured binocularly and monocularly with each eye while the non-tested eye was occluded with an eye patch.

### Data Analysis

During data recording, the quick CSF was initialized with a uniform prior. After data collection, all data sets were rescored with a more informative population prior (Dorr et al., 2017). As a summary statistic of binocular summation, we calculated the ratio of contrast sensitivity of the binocular to that of the dominant eye (AULCSF_Binocular_/AULCSF_DE_). For a finer-level analysis, we used the probability summation model, which is the simplest and most commonly used account in vision and hearing science (Dubois et al., 2013), as a theoretical yardstick. First, observers’ thresholds for the NDE, DE, and binocular viewing conditions were each converted into the probability of detecting a target signal given the assumed psychometric function (Pelli, 1987; Klein, 2001; Dubois et al., 2013; Equation 1). This function *P*(*c, τ*) describes the probability of a correct response as a function of signal contrast *c* and threshold contrast *τ*:

(1) $$P(c,\tau )=\gamma +(1-\gamma -\lambda )\times \text{ϕ}\left(\frac{0.6}{\beta }\times (c-\tau )\right)$$

where *γ* is guessing rate (= 0.1 for this 10-AFC paradigm), λ is the lapse rate, *β* is the slope of *P* (here, *β* = 0.25), and *Φ* is the cumulative distribution function of the normal distribution. This conversion was made as a function of spatial frequency and the final probability for each viewing condition (non-dominant eye *P*_NDE_, dominant eye *P*_DE_, and binocular viewing *P*_Binocular_) was the mean probability across spatial frequencies. Next, we computed the expected probability summation derived from the probabilistic summation of monocular signals from the non-dominant and dominant eyes as shown in Equation 2.

(2) $$P_{\text{Expected Binocular}}=P_{\text{NDE}}+P_{\text{DE}}-(P_{\text{NDE}}*P_{\text{DE}})$$

where *P* is the probability of detecting a target signal (corrected for the guessing rate of 10% by subtracting 0.1 from each term on the right-hand side and dividing by 0.9; for the left-hand side, this correction was inverted). We then compared this expected probability summation value *P*_Expected Binocular_ to the probability of binocular viewing condition *P*_Binocular_.

The posterior of the quick CSF provides a probability distribution over possible CSFs and their agreement with the data. We used the width of the credible interval, which encompasses 68.3% of the data, as a proxy to test-retest variability (Hou et al., 2016).

For statistical tests, we used a significance level of 0.05. Because a *p*-value tells us only how probable the observed outcome would be under the null hypothesis, but nothing about the relative probabilities for the null and H_1_, we also report the false positive risk (FPR) for an assumed uniform prior (Colquhoun, 2014). For example, consider the comparison of summary statistic AULCSF between non-dominant eyes of the AMB subgroup (observed distribution of mean = 0.99, SD = 0.467) and NSC eyes (mean = 1.65, SD = 0.147). We then sampled 100,000 population samples (*n* = 11, the number of amblyopes) from normal distributions D_AMB_ and D_NSC_ with these parameters, and calculated how often the originally observed *p*-value of approximately 0.0009 would occur when comparing the observed distribution of NSC eyes against data under the null hypothesis (M_NSC_ = 3 times; note that this is different from the *p*-value *100,000 = ~90 simulations where the effect size was at least as big as the originally observed effect) or a hypothesized true effect (M_AMB_ = ~1,000). The ratio M_NSC_/(M_NSC_+M_AMB_), which is influenced by statistical power of the experiment and the observed effect size and *p-value*, then gives us the FPR that we would see if the observed outcome was due to the null hypothesis being true; here, FPR = 0.003.

## Results

Data are publicly available at https://dataverse.harvard.edu/dataset.xhtml?persistentId=doi:10.7910/DVN/3XWZUN.

### Method Validation

The mean time to complete each quick CSF test was 170 (± 34) s, see Supplementary Figure S1. There was no effect of age on the reliability of quick CSF measurements, see Supplementary Figure S2.

### Monocular Contrast Sensitivity Deficit in Amblyopia

We first analyzed the four parameter values of the quick CSF and its two summary estimates, AULCSF and CSF Acuity for the three groups.

**Figure 1:**
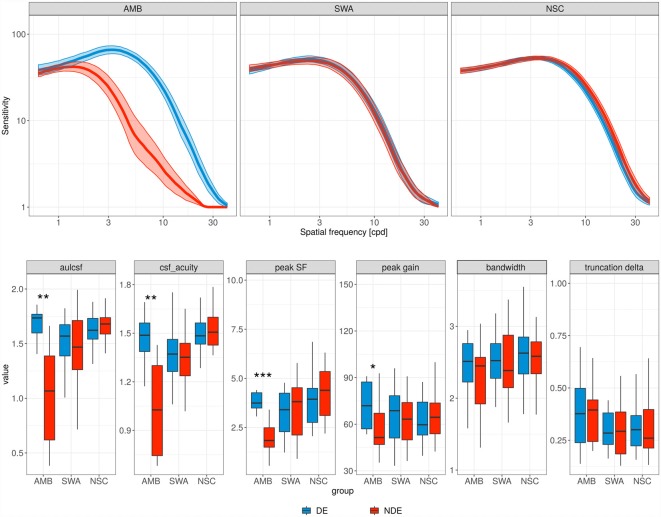
Contrast sensitivity functions (CSFs). Top row, each panel contains the mean CSF of the non-dominant eye (red curve) and the dominant eye (blue curve) for the different subject groups. AMB, amblyopia; SWA, strabismus without amblyopia; NSC, normally sighted controls. Shaded areas represent ±1 standard error of the mean (SEM). Bottom row, parameter distributions of the observed CSFs. Boxplot midlines indicate median values; “*”, “**,” and “***” denote statistical significance (two-sided Wilcoxon test) at the alpha level of 0.05, 0.01 and 0.001, respectively.

The contrast sensitivity deficits of AMB patients can be seen in Figure 1, which shows average CSFs over the different groups, as well as boxplots of the CSF parameter distributions: the CSF for the non-dominant eye (red curve) is diagonally shifted downward and to the left of the dominant eye (blue curve).

More specifically, the AMB group showed a significant reduction in peak SF (from 3.89 to 1.94 cyc/deg; *p* = 0.001, Wilcoxon test; FPR = 0.002) and peak gain (from 1.89 to 1.77 log10 sensitivity; *p* = 0.032; FPR = 0.146) for the non-dominant eye. However, no significant difference was observed in bandwidth and in low SF truncation (*p* = 0.32 and *p* = 0.64, respectively), and SWA and NSC did not show a significant difference between the two eyes for any of the parameters.

The changes in peak SF and peak gain for the AMB group resulted in a pronounced AULCSF deficit in the non-dominant (but not dominant) eye relative to the NSC group (mean 1.68 and 0.99 vs. 1.65 log10 units; *p* = 0.608 and *p* ≪ 0.001 (FPR = 0.003), respectively; two-sided *t*-test). AULCSF and CSF Acuity were also significantly lower in the non-dominant relative to the dominant eye (*p* < 0.01; FPR = 0.011 and 0.032, respectively) of AMB.

### Binocular Contrast Summation

**Figure 2:**
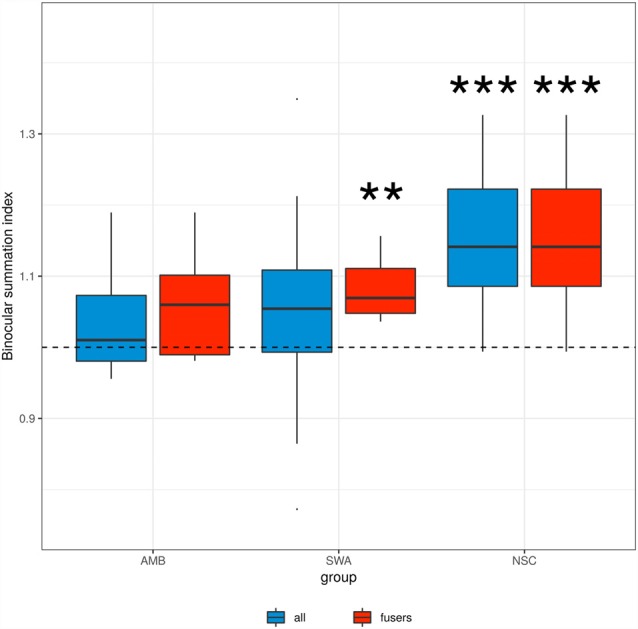
Binocular summation index. Binocular summation was evaluated as the ratio of contrast sensitivity of binocular vision to that of the dominant eye (AULCSF _Binocular_/AULCSF_DE_). Blue boxes indicate the data from all subjects in each group, red boxes indicate the data from the subset of amblyopic and strabismic subjects who are able to fuse at the testing distance. “**” and “***” denote statistical significance (two-sided *t*-test) at the alpha level of 0.01 and 0.001, respectively. SWA subjects who were able to fuse and NSC subjects showed an index significantly greater than 1, i.e., binocular summation.

Figure 2 shows binocular summation index distributions. While NSC subjects show evidence of binocular contrast summation (*p* ≪ 0.001, *t*-test; FPR ≪ 0.001), there was no such evidence for AMB (*p* = 0.14) or SWA (*p* = 0.09), when all subjects were included in the analysis (blue boxes).

In principle, a lack of binocular summation may be due to disparate retinal correspondence that resulted from ocular misalignment of the strabismic vision rather than an absence of neural summation (Thorn and Boynton, 1974). Thus, we looked at those patients who were able to fuse their eyes at the testing distance. As shown by the red boxes in Figure 2, there remained a lack of binocular contrast summation for this AMB subgroup (*p* = 0.17). The summation index was significantly greater than 1 in SWA subjects who were able to fuse (*p* < 0.003, *t*-test; FPR = 0.013). These results suggest that in strabismus, the lack of binocular summation is a direct consequence of ocular misalignment, whereas in amblyopia there is an additional fundamental developmental deficit in binocular vision.

### Understanding the Mechanism of Binocular Combination

It has been shown that the degree of binocular summation is greatly diminished with increasing interocular sensitivity difference (Marmor and Gawande, 1988; Pardhan and Gilchrist, 1990; Cagenello et al., 1993; Pardhan, 1993; Pineles et al., 2011) and depends on spatial frequency (Pardhan, 1996). We, therefore, compared the level of binocular contrast summation observed with the prediction of a probability summation model for independent sensory inputs.

We converted contrast sensitivity for the binocular condition of each observer to contrast detection threshold (probability correct 0.53) at 1,000 spatial frequencies (0.64~41 cpd). Based on (Equation 1), we computed the probability of a correct response at that contrast in the monocular conditions. These probabilities were averaged across spatial frequencies. Lastly, we computed the expected binocular detection probability based on the probability summation of the monocular detection probabilities (Equation 2).

**Figure 3:**
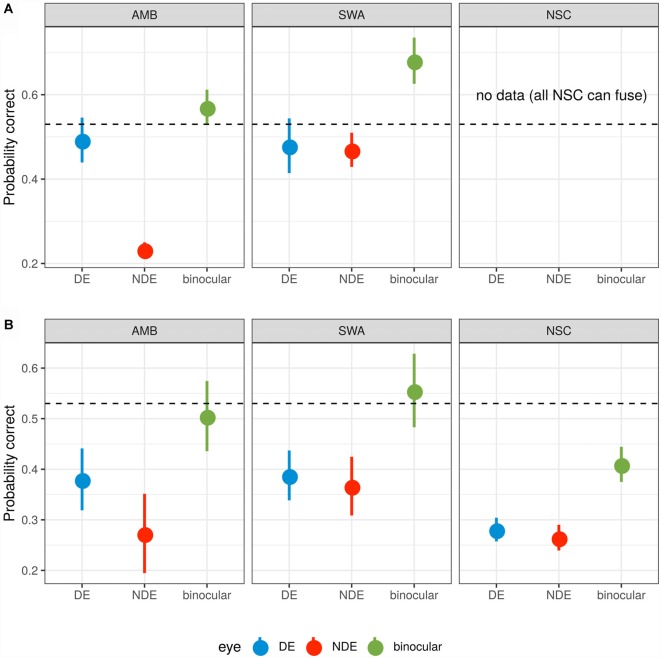
Probability summation. **(A)** Subjects who were unable to fuse the two monocular images. **(B)** Subjects who were able to fuse. The dotted line indicates 53% threshold for binocular target identification. For the average contrast needed to reach this binocular threshold, monocular detection probabilities are shown for the non-dominant (red) and the dominant (blue) eye. Based on probabilistic summation of each eye’s target detection probability, the expected probability for the binocular condition is shown in green. A value below the dotted line indicates that actual binocular summation exceeds probabilistic summation. A value above the dotted line indicates that the monocular images inhibit one another so that performance is worse than expected from target detection by independent monocular mechanisms. Error bars represent ±1 SEM.

Data points in Figure 3 show the probability of target detection in each viewing condition. Dotted lines indicate the observed binocular target detection probability (0.53). As expected, AMB exhibited considerable difference in target detection probability between both eyes (*p* < 0.01, Wilcoxon test; FPR = 0.079) while SWA and control groups did not show any significant differences between the two eyes (all *p* > 0.05).

For AMB subjects (with and without the ability to fuse the monocular images) and those SWA subjects that were able to fuse (Figure 3 top left and bottom row, left and center), the observed binocular contrast summation did not differ from that predicted from simple probability summation (all *p* > 0.05, *t*-test). Binocular summation of NSC subjects, however, was significantly greater than predicted from the probability summation (*p* < 0.008; FPR = 0.020). For the subset of SWA subjects who were unable to fuse (Figure 3A center), this pattern reversed and binocular summation was significantly impaired relative to probability summation (*p* < 0.02; FPR = 0.088).

### Relationship Between Visual Acuity and CSF Parameters

There were significant correlations between logMAR acuity and quick CSF measures both for the non-dominant eye and for interocular differences with *r*^2^ values between 0.54 and 0.65 (all *p* ≪ 0.001). Variability of logMAR acuity can be well-accounted for by log CSF Acuity (*r*^2^ = 0.64). Slightly less accountability (*r*^2^ = 0.55) was observed in the regression of logMAR acuity on AULCSF (see Supplementary Figure S3).

## Discussion and Conclusion

Amblyopia is associated with anomalies in contrast sensitivity (Howell et al., 1983; McKee et al., 2003). Consistent with earlier findings, the present study demonstrates that individuals with amblyopia show a significant loss of contrast sensitivity in the non-dominant eye while the CSF of the dominant eye appears to be normal. By examining the CSF parameters, we show that the overall reduction in the CSF of the amblyopic eye was largely explained by significantly reduced peak spatial frequency and gain in the non-dominant eye. Because bandwidths stayed the same, sensitivity was lost particularly at high spatial frequencies. When we examined the monocular CSFs of SWA patients, we found no such differences in their monocular CSFs.

We also measured the deficits in binocular contrast sensitivity in AMB and SWA subjects. The superiority of binocular over monocular viewing is well documented in normal vision (Campbell and Green, 1965; Blake and Fox, 1973; Thorn and Boynton, 1974; Legge, 1984a). Binocular summation is often quantified as the ratio of binocular sensitivity to monocular sensitivity. While log probability summation for two equally detectable signals is approximately a factor of 1.2 (Tyler and Chen, 2000), many studies have shown that binocular contrast sensitivity is approximately 40% greater than monocular sensitivity (Legge, 1984b; Anderson and Movshon, 1989; Tyler and Chen, 2000; Meese et al., 2006; Baker et al., 2007b). Our NSC results are in good agreement with these estimates of binocular summation. Because this binocular performance enhancement exceeds the expected improvement from probability summation alone, it has been believed that this enhancement likely reflects neural summation (Campbell and Green, 1965; Blake and Fox, 1973; Bacon, 1976; Legge, 1984b; Anderson and Movshon, 1989).

Binocular contrast summation diminishes as interocular sensitivity difference increases (Marmor and Gawande, 1988; Pardhan and Gilchrist, 1992; Jiménez et al., 2004; Pineles et al., 2013, 2015a). Thus, without compensating for the difference in sensitivity between the two eyes, binocular contrast summation in amblyopia is either absent or greatly compromised (Levi et al., 1979, 1980; Pardhan and Gilchrist, 1992; Baker and Meese, 2007). Consistent with previous findings for binocular acuity summation (Jiménez et al., 2004), our results confirmed the lack of binocular contrast summation (Lema and Blake, 1977; Levi et al., 1980; Sireteanu et al., 1981; Hess et al., 2014) in AMB subjects. We further show that binocular contrast summation is impaired in those SWA subjects who were unable to fuse at near distances; SWA subjects who were able to fuse, on the other hand, did exhibit significant binocular summation. These findings are in good agreement with studies showing that binocular acuity summation is greater in subjects with greater control over intermittent exotropia (Yulek et al., 2017) and that strabismus surgery to align the eyes can lead to improvements in binocular vision (Pineles et al., 2015a; Kattan et al., 2016; Chang et al., 2017).

We also used the monocular and binocular psychometric functions to estimate probability summation. The results confirmed the analyses of dominant eye and binocular AULCSF; the binocular contrast sensitivity of AMB or SWA subjects who were able to fuse was consistent with simple probability summation between independent detectors. This indicates an impairment of binocular contrast vision. Moreover, for SWA subjects who were unable to fuse at near distances, binocular contrast sensitivity was worse than expected from probability summation, suggesting an inhibitory process that impairs the combination of monocular sensitivity. These results are in good agreement with previous studies showing inhibition of binocular acuity summation in strabismic observers (Pineles et al., 2013, 2014a).

The use of the probability summation model allowed us to evaluate mechanisms of binocular interactions based on monocular and binocular CSFs when the two eyes have drastically different sensitivities. The method can be extended directly to many other visual conditions, such as age-related macular degeneration (AMD), glaucoma, and cataract. It can also be extended to other measures of visual function, such as monocular and binocular visual acuity, and monocular and binocular perimetry.

In conclusion, our results suggest that monocular and binocular contrast sensitivity deficits define important characteristics of amblyopia and strabismus that are not captured by visual acuity alone. Furthermore, our results identify a key role of fusion in binocular summation. Finally, measurement of both the monocular and binocular CSFs was possible rapidly and reliably even in young children, which may allow clinicians to more accurately assess individual patients’ functional contrast sensitivity and acuity outcomes and prognosis.

## Data Availability

The datasets generated for this study are available on request to the corresponding author.

## Ethics Statement

This study was carried out in accordance with procedures approved by the IRB of Boston Children’s Hospital with written informed consent from all subjects or (in addition to subjects’ assent) from the parents or legal guardian of subjects aged <18 years. All subjects gave written informed consent in accordance with the Declaration of Helsinki. The protocol was approved by the IRB of Boston Children’s Hospital.

## Author Contributions

MYK, MK, KC, DH, Z-LL, and PB contributed to the conception and design of the study. AM collected the data and organized the database. MD, MYK, and LL analyzed the data. MD and PB wrote sections of the manuscript. All authors contributed to manuscript revision, read and approved the submitted version.

## Conflict of Interest Statement

MD, LL, Z-LL, and PB have intellectual property in quick CSF assessment and equity in Adaptive Sensory Technology (AST), a company that aims to commercialize the quick CSF; LL also holds employment at AST. The remaining authors declare that the research was conducted in the absence of any commercial or financial relationships that could be construed as a potential conflict of interest.
