# Supplementary material for: Binocular Summation and Suppression of Contrast Sensitivity in Strabismus, Fusion and Amblyopia
Source: Front Hum Neurosci. 2019 Jul 12;13:234. doi: 10.3389/fnhum.2019.00234 (PMC6640006; doi:10.3389/fnhum.2019.00234)
Supplement: Supplementary file 1 [file Data_Sheet_1.PDF]

Supplementary material for Dorr et al., "Binocular Summation and Suppression of Contrast Sensitivity in Strabismus, Fusion and Amblyopia"

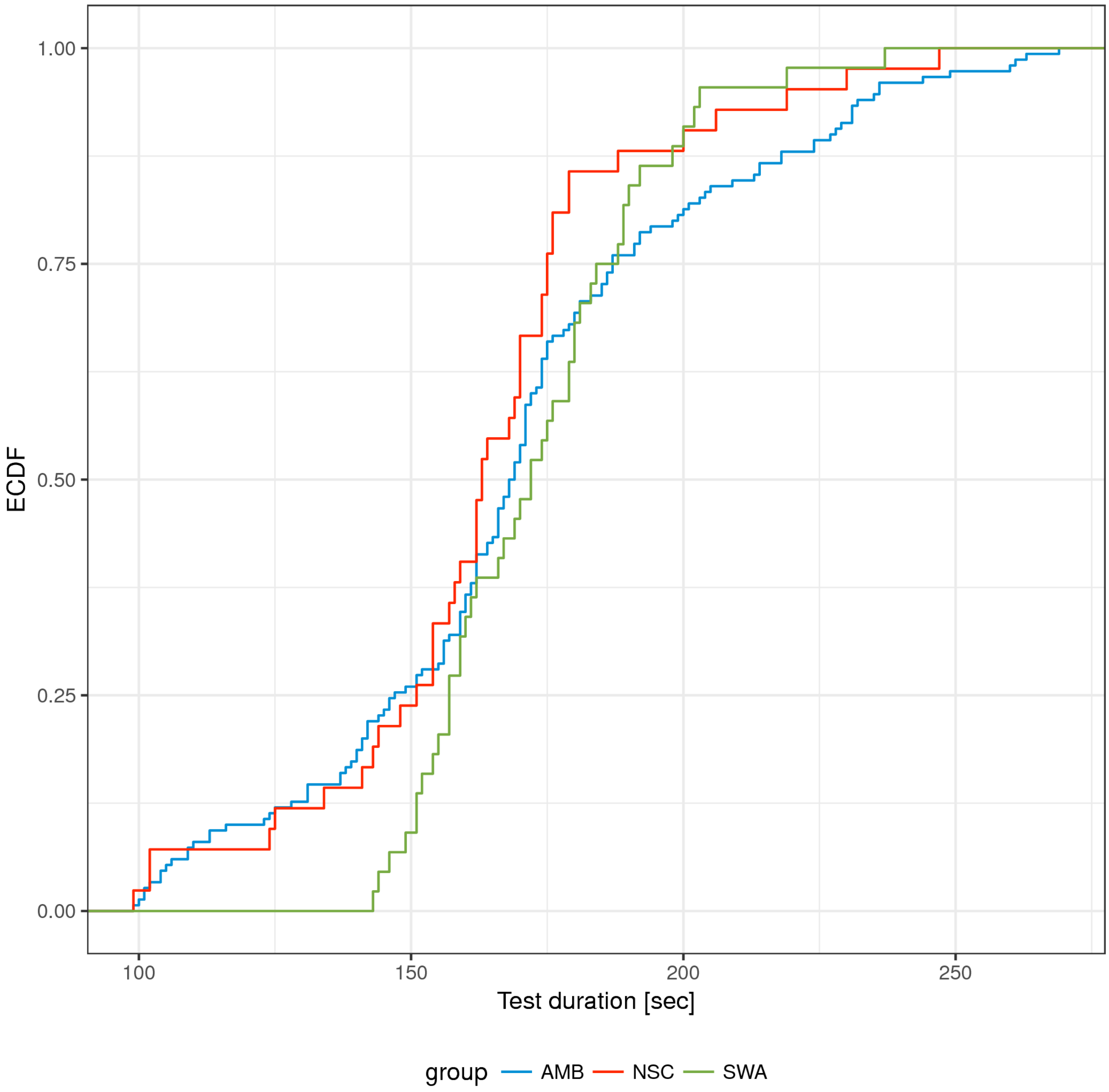

Supplementary Figure S1: Empirical cumulative distribution functions of test durations.

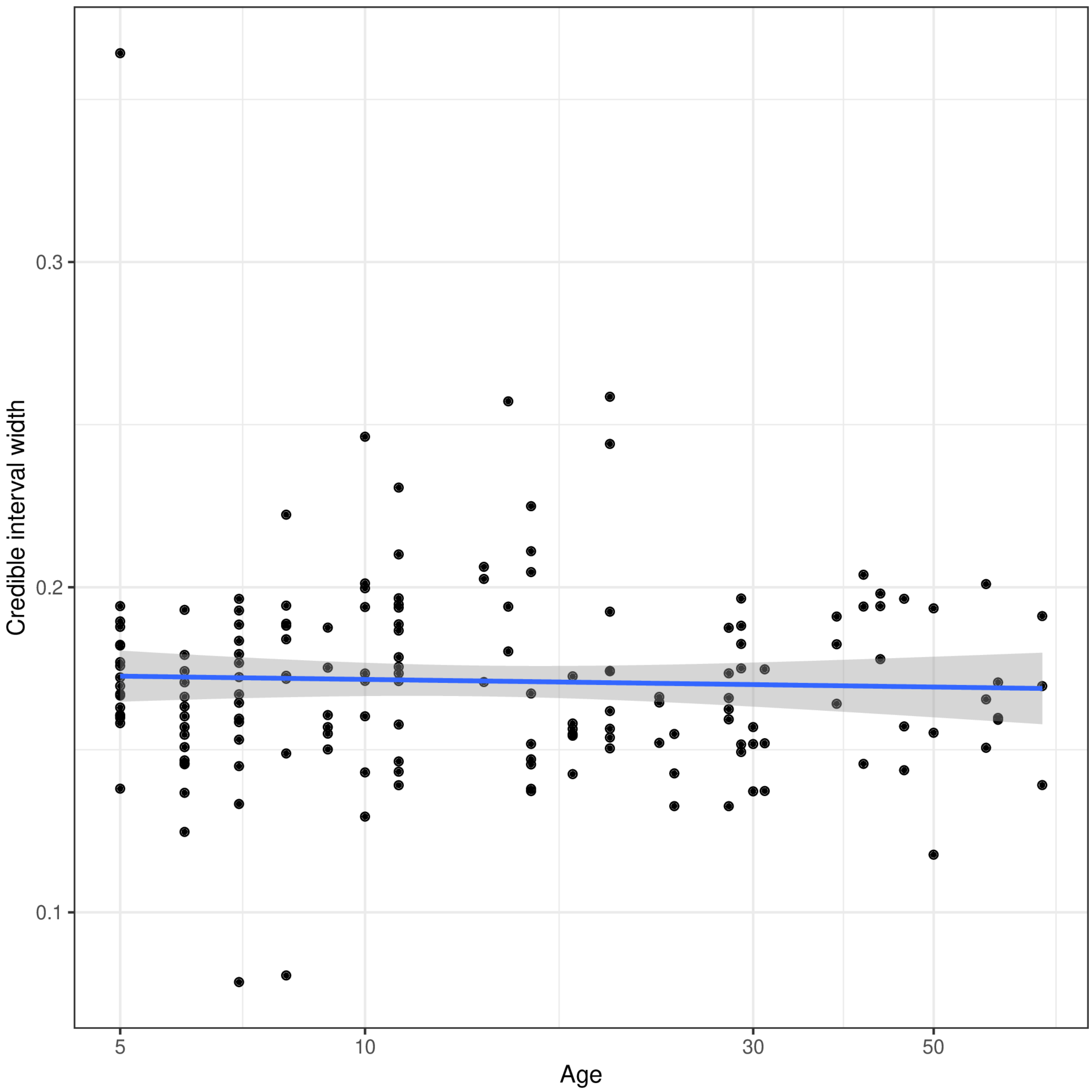

Supplementary Figure S2. Reliability of quick CSF measurements as a function of age. There was no effect of age on the width of the credible interval of the posterior estimate of the area under the log CSF, a summary statistic of visual function.

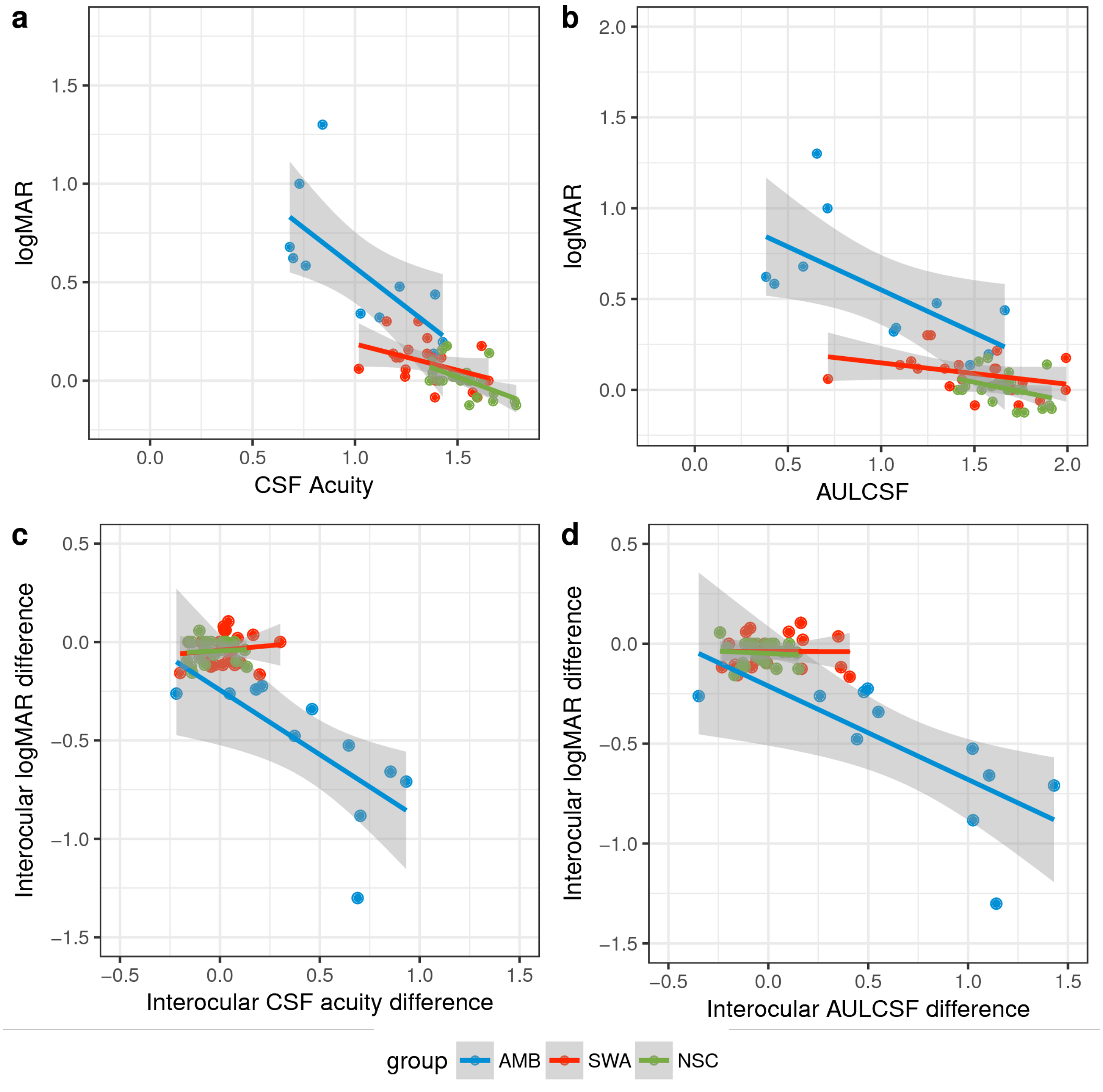

Supplementary Figure S3. Relationships between logMAR visual acuity and quick CSF. a) logMAR acuity plotted against log CSF Acuity for the non-dominant eyes of subjects with amblyopia (blue), strabismus without amblyopia (red) and normally-sighted controls (green); **b)** logMAR acuity plotted against AULCSF for the non-dominant eyes of all participants. **c)** Interocular logMAR acuity difference plotted as a function of interocular log CSF Acuity difference; **d)** Interocular logMAR acuity difference plotted as a function of interocular AULCSF difference. Each data point represents an individual subject. Shaded areas represent a 95% confidence interval for the slope of the regression line. Interocular differences in logMAR acuity were very small by design for the two non-amblyopic groups, so that the red and green regression lines in the bottom row are very flat.
